# Sex-Specific Risk Factors for Dynapenia in Korean Middle-Aged and Older Adults: A Cross-Sectional Study Based on the Korea National Health and Nutrition Examination Survey 2014–2019

**DOI:** 10.3390/jpm15110507

**Published:** 2025-10-25

**Authors:** Hyunjae Yu, Hye-Jin Kim, Heeji Choi, Chulho Kim, Jae Jun Lee

**Affiliations:** 1 Research Institute, Neurophet Inc., Seoul 06234, Republic of Korea; yhj93@neurophet.com; 2 Institute of New Frontier Research Team, Hallym University College of Medicine, Chuncheon 24252, Republic of Korea; 43786@hallym.ac.kr; 3 Artificial Intelligence Research Center, Hallym University Sacred Heart Hospital, Chuncheon 24253, Republic of Korea; choiheeji@hallym.or.kr; 4 Department of Neurology, Hallym University College of Medicine, Chuncheon 24252, Republic of Korea; 5 Department of Anesthesiology, Hallym University College of Medicine, Chuncheon 24252, Republic of Korea

**Keywords:** muscle weakness, muscle strength, sex characteristics, data mining, aging

## Abstract

**Background/Objectives**: Dynapenia, characterized by an age-related decline in muscle strength, has recently gained attention as a major public health concern. While prior studies identified individual risk factors, little is known about how these factors cluster differently by sex. This study investigated sex-specific risk factors and their combinations associated with dynapenia among Korean middle-aged and older adults. **Methods**: We analyzed 22,850 participants aged ≥ 40 years from the 2014–2019 Korea National Health and Nutrition Examination Survey. Dynapenia was defined as handgrip strength < 28 kg in men and <18 kg in women. Sex-stratified multivariable logistic regression identified independent predictors, and association rule mining (ARM) detected synergistic risk factor combinations. **Results**: Dynapenia was more prevalent in women (13.9%) than in men (8.5%). Advancing age, physical inactivity, lack of resistance exercise, and a high incidence of diabetes and stroke were consistent risk factors in both sexes. However, ARM revealed distinct clustering patterns: behavioral factors predominated in men, whereas socioeconomic disadvantage and metabolic comorbidities were more relevant in women with dynapenia. **Conclusions**: These findings emphasize the need for sex-specific prevention strategies for dynapenia, promoting resistance exercise among men and addressing both inactivity and socioeconomic barriers in women.

## 1. Introduction

Dynapenia, defined as the age-related loss of muscle strength [1,2], has emerged as a significant public health concern in aging societies. Unlike sarcopenia, which primarily refers to the decline in muscle mass, dynapenia especially emphasizes the deterioration of muscle strength [1,2]. It has been recognized as a more reliable indicator of functional limitations, disability, and mortality in older adults [3,4].

Dynapenia becomes more prevalent with age and is shaped by lifestyle behaviors, socioeconomic status, and chronic health conditions [5]. Cross-national data show wide –heterogeneity—approximately 17–28%in Brazil [6], 27–34% in Mexico [7], and up to 44% in U.S cohorts [8]—largely reflecting differences in definitions, measurement protocols, and population structure. Large cohorts such as the UK Biobank [9] and the U.S Health and Retirement Study [8] also demonstrate marked sex differences in both prevalence and determinants. In Korea, analyses of the Korea National Health and Nutrition Survey (KNHANES) have reported significant sex differences in the prevalence and determinants of dynapenia [10]. These sex-specific patterns may be explained by biological, behavioral, and social factors [11,12]. For example, differences in physical activity levels, participation in resistance exercise, alcohol consumption, smoking habits, and the prevalence of chronic diseases have been proposed as potential contributors [13].

However, most previous studies have examined these risk factors in isolation, without considering how they might interact with each other. In real-world settings, health risk factors tend to cluster together, and their combined effects may be synergistic rather than additive [14]. Therefore, it is crucial to identify such combinations when developing tailored prevention and intervention strategies, particularly when patterns differ between men and women.

Association rule mining (ARM) is a data-driven approach that can identify frequent and clinically relevant combinations of variables within large datasets [15]. This method provides complementary insights to those provided by traditional regression models [16]. By combining multivariable logistic regression with ARM, it is possible to estimate the independent associations between risk factors and dynapenia, while also exploring complex patterns of co-occurring risk factors that may inform targeted, sex-specific health policies.

Therefore, this study aimed to investigate sex-specific patterns of risk factor combinations associated with dynapenia in Korean middle-aged and older adults. Using nationally representative data from KNHANES (2014–2019), we applied multivariable logistic regression and ARM to identify independent predictors of dynapenia, as well as synergistic combinations of lifestyle, socioeconomic, and clinical risk factors.

## 2. Materials and Methods

### 2.1. Study Design and Data Source

This cross-sectional study used data from the KNHANES. The KNHANES is a nationwide, population-based survey of non-institutionalized Korean civilians conducted by the Korea Centers for Disease Control and Prevention. It employs stratified, multistage, clustered probability sampling design to ensure adequate representation of the Korean population. The survey consists of three components: a health interview, a health examination and a nutrition survey. The health interview and examination are conducted by trained medical staff at mobile examination centers, while the nutrition survey is carried out thorough home visits by dietitians. All participants provided written informed consent. The KNHANES protocol was approved by the Institutional Review Board of the Korea Centers for Disease Control and Prevention, and all data are publicly available through the official KNHANES portal (https://knhanes.kdca.go.kraccessed on 4 Mar 2025) following user registration and institutional approval [17].

### 2.2. Participants

**Figure 1:**
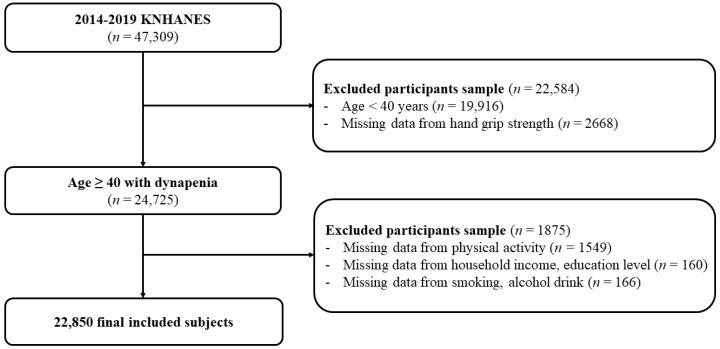
Flow chart of the study participants. KNHANES, Korean National Health and Nutrition Examination Survey.

Among the 47,309 participants in the 2014–2019 KNHANES, we excluded individuals younger than 40 years (*n* = 19,916) and those with missing handgrip strength measurements (*n* = 2668). Additional exclusions were made for individuals with missing information on physical activity, socioeconomic status, or lifestyle factors. The final analytical sample consisted of 22,850 participants (Figure 1). To assess potential selection bias, we compared characteristics before and after listwise deletion (Supplementary Table S1).

The study protocol and data collection procedures were approved by the Institutional Review Board of the KNHANES or affiliated research institutions (IRB No.: 2025-08-004).

### 2.3. Definition of Variables

The Sociodemographic variables included age, categorized into three groups based on modified World Health Organization age classification [18]: 40–59 years, 60–74 years, and ≥75 years. Household income was divided into quartiles [19], and educational level was classified as ≤high school versus ≥college.

Lifestyle factors were assessed using standardized questionnaires. Smoking status was classified as current smoker or non-smoker, and alcohol consumption was defined as high-risk drinking (≥7 drinks per occasion for men and ≥5 drinks per occasion for women, at least twice per week) or low-risk drinking [19]. Physical activity was categorized as <150 min per week or ≥150 min per week of moderate-to-vigorous physical activity [20]. Resistance exercise was defined as performing muscle-strengthening activity on ≥2 days per week or <2 days per week [20].

The chronic diseases included hypertension, diabetes, dyslipidemia, ischemic heart disease, stroke, cancer, and arthritis. All disease variables were based on self-reported information from the KNHANES.

### 2.4. Muscle Strength Measurement

Muscle strength was measured using a digital grip strength dynamometer (T.K.K 5401, Takei Scientific Instruments Co., Ltd., Tokyo, Japan). Each hand was tested three times in an alternating manner, for a total of six trials. The highest value was recorded as the final handgrip strength. Dynapenia was defined, according to the 2019 Asia Working Group for Sarcopenia (AWGS) criteria as a handgrip strength of <28 kg in men and <18 kg in women [21].

### 2.5. Statistical Analysis

#### 2.5.1. Baseline Characteristics and Logistic Regression Analysis

Descriptive statistics were used to analyze the baseline characteristics of the participants, stratified by dynapenia status and sex. All variables were categorical and presented as frequencies and percentages. Differences between groups were compared using the chi-squared test and standardized differences were additionally calculated to quantify effect sizes (Supplementary Table S2).

To identify factors associated with dynapenia, univariable and multivariable logistic regression analyses were conducted separately for each sex. In the univariable analysis, each covariate was tested individually for its association with dynapenia, and results were expressed as odds ratios (ORs) with 95% confidence intervals (CIs). Multivariable logistic regression models were then constructed. Model 1 was adjusted for age only, while Model 2 was adjusted for age, household income, education level, smoking status, alcohol consumption, physical activity, resistance exercise, hypertension, diabetes, dyslipidemia, ischemic heart disease, stroke, cancer, and arthritis. As a sensitivity analysis, complex-sample logistic regression models accounting for stratification, clustering, and sampling weights were also performed in accordance with the KNHANES analytic guidelines (Supplementary Table S3) and dynapenia was alternatively defined using dominant hand grip strength only. Statistical significance was set at *p* < 0.05. All analyses were performed using R statistical software (version 4.5.1).

#### 2.5.2. Association Rule Mining

Association rule mining (ARM) was employed to identify synergistic combinations of risk factors associated with dynapenia. Among the available ARM approaches, the Apriori algorithm [22] was selected for its effectiveness in identifying frequent item sets. The analysis was performed separately for men and women to identify sex-specific patterns. Only variables with a prevalence of at least 15% in the study population, along with the binary dynapenia outcome, were included as covariates (‘items’) in the mining process. The dichotomization criteria for all categorical variables used in the ARM analysis are summarized in Supplementary Table S4. An association rule was expressed in the form {A} → {B}, where {A} represents a set of antecedent items (risk factors) and {B} denotes the consequent item (presence of dynapenia).

To ensure clinical relevance and statistical robustness, a minimum support threshold of 1% was applied, as support was considered the most important criterion for identifying clinically meaningful patterns. Although confidence was calculated for each rule, no minimum confidence threshold was applied in the final selection. Instead, rules were ranked by their lift values, with those exhibiting the highest lift selected for interpretation. Lift was defined as the ratio of the observed co-occurrence of antecedent and consequent to that expected under independence, with values greater than one indicating a positive association [23]. Detailed algorithm parameters applied in this study, including support, confidence, and lift thresholds, are provided in Supplementary Table S5. All ARM analyses were implemented using the arules package (version 1.7-11) in R statistical software (version 4.5.1).

## 3. Results

### 3.1. Participant Characteristics

This study included 22,850 participants (9951 men and 12,899 women). The prevalence of dynapenia was 8.5% in men (*n* = 843) and 13.9% in women (*n* = 1788). As shown in Table 1, the prevalence of dynapenia increased significantly with age in both sexes (*p* < 0.001). Among participants aged ≥ 75 years, 56.3% of men and 44.7% of women had dynapenia.

Socioeconomic factors were strongly associated with dynapenia, with low household income (*p* < 0.001) and lower educational attainment (*p* < 0.001) being significantly more common among participants with dynapenia. Lifestyle factors also demonstrated significant associations, including physical activity of <150 min per week and resistance exercise performed less than twice per week. In addition, dynapenia was significantly more prevalent among participants with chronic diseases such as diabetes, hypertension, stroke, ischemic heart disease, and arthritis (all *p* < 0.001).

### 3.2. Sex-Specific Risk Factors for Dynapenia

Table 2 presents the results of the multivariable logistic regression analysis of factors associated with dynapenia, stratified by sex. Age was the strongest predictor of dynapenia in both men and women, with significantly higher odds observed in individuals aged ≥ 60 years compared with the reference group (40–59 years). Higher household income and educational attainment were inversely associated with dynapenia, with lower odds among participants in the higher income and education groups.

### 3.3. Association Rule Mining for Dynapenia Risk Patterns

Among men, engaging in ≥150 min of moderate-intensity physical activity per week (OR, 0.63; 95% CI, 0.53–0.75) and performing resistance exercise at least twice per week (OR, 0.47; 95% CI, 0.38–0.59) were both significantly associated with a reduced risk of dynapenia. In contrast, diabetes (OR, 1.23; 95% CI, 1.00–1.50) and a history of stroke (OR, 2.06; 95% CI, 1.55–2.73) were associated with an increased risk. Other chronic diseases, including hypertension, dyslipidemia, ischemic heart disease, and cancer, did not show significant associations after full adjustment.

A similar pattern was observed in women. Higher levels of physical activity (OR, 0.68; 95% CI, 0.60–0.78) and resistance exercise (OR, 0.58; 95% CI, 0.47–0.71) were protective, whereas diabetes (OR, 1.39; 95% CI, 1.20–1.62) and stroke (OR, 1.58; 95% CI, 1.23–2.05) were positively associated with dynapenia. Notably, arthritis showed a significant association with dynapenia in women (OR, 1.16; 95% CI, 1.03–1.31), but not in men. When dynapenia was defined based on grip strength of the dominant hand, the results remained consistent with those obtained using the maximum value from both hands (Supplementary Table S6).

**Figure 2:**
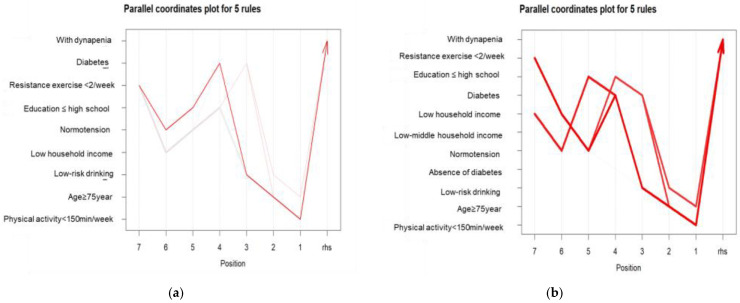
Parallel coordinates plots of the top five association rules for dynapenia risk factors by sex: (**a**) Men and (**b**) Women. Each line represents one of the top five association rules with the highest lift values. The red line shows the strongest rule for each sex, i.e., the rule with the highest lift value. The intensity of the red color indicates the strength of the association rule, with a darker shade signifying a stronger association. Variables are arranged along the parallel axes, with the position on each axis indicating the presence or absence of specific risk factors. And, the direction of the arrow represents a reverse association, implying inference from the outcome back to its potential antecedent conditions. The lines converge toward “With dynapenia”, highlighting the distinct patterns of risk factor combinations associated with this condition.

**Figure 3:**
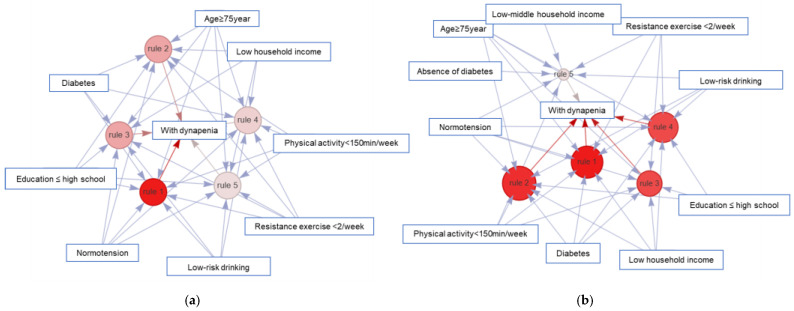
Network visualization of the top five association rules for dynapenia by sex: (**a**) Men and (**b**) Women. Red nodes indicate dynapenia outcomes. Numbered nodes (rules 1–5) represent the top- ranked association rules, with node size corresponding to lift values. Blue arrows indicate directional associations from antecedent conditions to outcomes. In men (**a**), the network shows clustering around behavioral factors, such as physical inactivity and lack of resistance exercise whereas in women (**b**), clustering is more prominent around socioeconomic factors (e.g., low household income) and metabolic comorbidity (presence or absence of diabetes).

Association rule mining identified distinct patterns of risk factor combinations associated with dynapenia, with notable sex-specific differences (Table 3; Figure 2 and Figure 3). In men, dynapenia was most frequently associated with the combination of older age (≥75 years), low physical activity (<150 min/week), and lack of resistance exercise (<2 times/week). These three variables consistently appeared across the top-ranked association rules. The rule {physical activity < 150 min/week, age ≥ 75 years, diabetes, education ≤ high school, resistance exercise < 2/week} → dynapenia demonstrated the highest lift value (lift = 6.42), indicating that the co-occurrence of these sociodemographic and behavioral risk factors substantially increased the likelihood of dynapenia beyond chance. Other frequent rules also included diabetes and low educational attainment, although these yielded relatively lower lift values compared with lifestyle-related behaviors.

In women, association rules more prominently featured advanced age (≥75 years), low physical activity (<150 min/week), low household income, and diabetes. These four risk factors consistently appeared across the top five rules. The rule {physical activity < 150 min/week, age ≥ 75 years, diabetes, low household income, resistance exercise < 2/week} → dynapenia exhibited a particularly high lift value (lift = 4.23), reflecting a clustering of socioeconomic disadvantage and inactivity in older women with dynapenia. Furthermore, although diabetes appeared with similar frequency in association rules for both sexes, its predictive impact was more pronounced in women. Rules containing diabetes consistently showed higher confidence levels (58–59%) compared with those without diabetes (54%), suggesting a stronger contribution of metabolic comorbidity to dynapenia among women. The list of top association rules with detailed metrics for both sexes is presented in Supplementary Table S7.

## 4. Discussion

Using multivariable logistic regression and ARM, we identified sociodemographic, lifestyle, and clinical factors that were independently associated with dynapenia in this nationally representative sample of Korean middle-aged and older adults, as well as sex-specific combinations of these factors. Advancing age was the strongest predictor of dynapenia in both sexes. Participants aged ≥ 75 years had markedly higher odds than those aged 40–59 years (OR = 16.90 in men, OR = 8.88 in women), reflecting the age-related decline in muscle strength observed in our population. These findings are consistent with previous studies [1,2] that have similarly reported aging as a major determinant of muscle weakness and functional decline. Regular physical activity and resistance exercise were protective factors [6], while diabetes and stroke [24] were detrimental, with notable sex-specific differences.

Interestingly, high-risk alcohol drinking showed an inverse association with dynapenia in our study. Similar findings have been reported in other epidemiological research [25,26], possibly reflecting the “healthy drinker effect”, or “sick quitter bias”, whereby healthier individuals are more likely to consume alcohol, while those in poor health tend to abstain or reduce their intake. However, without direct measures of functional status or longitudinal data to identify former drinkers who quit due to poor health, we cannot fully exclude residual confounding by unmeasured health status. Given the cross-sectional design of the study and the reliance on self-reported alcohol intake, these findings should be interpreted with substantial caution and not be construed as evidence of a protective effect of alcohol on muscle strength. Further longitudinal studies incorporating detailed drinking trajectories and comprehensive functional assessments are warranted to clarify this relationship, as residual confounding and reporting bias cannot be ruled out.

Multivariable regression analysis revealed an inverse association between higher household income, educational level, and dynapenia. This supports existing evidence that socioeconomic status influences muscle strength by improving access to health resources and physical activity opportunities [27,28]. Among men, those in the highest income quartile had 65% lower odds of dynapenia (OR, 0.35; 95% CI, 0.26–0.47), while those who had attended college had 31% lower odds (OR, 0.69; 95% CI, 0.54–0.88). Similar patterns were observed in women, though the protective effect of education was attenuated following full adjustment. Meeting the WHO’s recommended physical activity levels and engaging in resistance exercise at least twice weekly were both associated with substantially lower odds of dynapenia. In men, adequate physical activity (OR, 0.63; 95% CI, 0.53–0.75) and resistance exercise (OR, 0.47; 95% CI, 0.38–0.59) provided strong protection. These findings are consistent with those of previous intervention and observational studies [6,29]. Women showed comparable protective effects (OR, 0.68 and 0.58), highlighting the importance of physical activity for both sexes. Diabetes (OR = 1.23 in men, OR = 1.39 in women) and stroke (OR = 2.06 in men, OR = 1.58 in women) were significant risk factors for both sexes. This is consistent with research linking metabolic and cerebrovascular diseases to muscle weakness via chronic inflammation, insulin resistance, and neuromuscular impairment [30,31]. Notably, arthritis was only associated with dynapenia in women (OR, 1.16; 95% CI, 1.03–1.31), suggesting sex-specific musculoskeletal pathways [31,32]. These findings also highlight important sex-specific pathways underlying dynapenia. In men, behavioral factors such as insufficient physical activity and lack of resistance exercise emerged as dominant contributors, whereas in women, socioeconomic disadvantage and metabolic comorbidities appeared to play a more prominent role. This contrast suggests that the mechanisms leading to dynapenia may differ by sex, with behavioral factors exerting greater influence in men and socioeconomic or clinical burdens being more critical in women. This aligns with recent evidence that composite phenotypes across the muscle–bone–adiposity axis show explicit sex-and age-specific differences, reinforcing our sex-specific ARM and the need for risk profiling [33].

Combining logistic regression and ARM emphasizes the importance of examining both independent associations and complex co-occurrence patterns [34]. While regression identifies modifiable predictors with independent effects, ARM reveals high-risk clusters with synergistic properties [35]. Together, these approaches enable the development of more targeted, sex-specific prevention strategies.

In our study, older Korean men were most likely to benefit from strategies that promote participation in resistance exercise. In contrast, interventions for women should focus on reducing physical inactivity and addressing socioeconomic barriers and chronic disease management. These findings support the development of risk stratification tools to assist healthcare providers in identifying high-risk individuals and improving the cost-effectiveness of dynapenia prevention programs. Moreover, the identified risk factor combinations could be incorporated into clinical decision support systems to guide personalized prevention strategies.

This study’s strengths include its use of a large, nationally representative dataset containing standardized handgrip strength measurements, as well as comprehensive socioeconomic, lifestyle, and clinical data. The dual analytical approach enhanced the robustness of the results, revealing clinically relevant patterns that would have remained undetected using regression analysis alone. The observed prevalence of dynapenia (8.5% in men and 13.9% in women) in consistent with that reported in other studies of Asian populations [36], thus supporting the study’s external validity. Nevertheless, several limitations warrant acknowledgement. The cross-sectional design precludes causal inference and cannot rule out reverse causality. Self-reported variables may introduce misclassification bias. Associations identified by ARM may be context-specific and require external validation. Furthermore, residual confounding from unmeasured variables, such as dietary intake, hormonal status, and inflammatory markers, cannot be ruled out.

The absence of dietary intake data and inflammatory/oxidative stress biomarkers represents an important limitation. Nutritional status and systemic inflammation are established determinants of muscle strength that likely correlate with several of our measured risk factors (e.g., chronic diseases, socioeconomic status). Their omission may have led to residual confounding, potentially overestimating the independent associations between lifestyle factors, chronic conditions, and dynapenia. Emerging evidence indicates that undernutrition in older adults clusters with elevated inflammatory markers and lower albumin/hemoglobin levels [37], while oxidative stress provides a plausible mechanistic link between inadequate nutrition and musculoskeletal decline [38]. Future studies should adopt longitudinal designs to clarify the temporal relationships between risk factors and dynapenia, and to evaluate the effectiveness of interventions targeting high-risk clusters. Integrating biomarker data, including inflammatory markers and hormonal profiles, with ARM could provide further insight into the biological pathways underlying the observed patterns. Additionally, research on social determinants and mechanistic studies investigating sex differences in dynapenia risk could inform precision medicine approaches to prevention and management. This could ultimately lead to more effective and individualized strategies for maintaining muscle strength in aging populations.

## 5. Conclusions

Dynapenia in Korean middle-aged and older adults was consistently associated with aging, physical inactivity, insufficient resistance exercise, diabetes, and stroke. ARM revealed sex-specific clustering, with behavioral factors predominating in men and socioeconomic and metabolic factors in women. These findings support tailored prevention strategies to preserve muscle strength and independence in aging populations.

## Figures and Tables

**Table 1:** Sex-specific descriptive characteristics of participants according to dynapenia status, presented as *n* (%).

	Men (*n* = 9951)			Women (*n* = 12,899)		
	Without Dynapenia (*n* = 9108)	Dynapenia (*n* = 843)	*p* Value	Without Dynapenia (*n* = 11,111)	Dynapenia (*n* = 1788)	*p* Value
Age						
40–59	5005 (55.0%)	86 (10.2%)	<0.001	6513 (58.6%)	359 (20.1%)	<0.001
60–74	3271 (35.9%)	282 (33.5%)		3694 (33.2%)	629 (35.2%)	
≥75	832 (9.1%)	475 (56.3%)		904 (8.1%)	800 (44.7%)	
Household income						
low	1551 (17.0%)	448 (53.1%)	<0.001	2302 (20.7%)	894 (28.0%)	<0.001
middle-low	2301 (25.3%)	215 (25.5%)		2790 (25.1%)	407 (22.8%)	
middle-high	2438 (26.8%)	113 (13.4%)		2835 (25.5%)	265 (14.8%)	
high	2818 (30.9%)	67 (7.9%)		3184 (28.7%)	222 (12.4%)	
Education						
<college	5807 (63.8%)	738 (87.5%)	<0.001	8353 (75.2%)	1634 (91.4%)	<0.001
≥college	3301 (36.2%)	105 (12.5%)		2758 (24.8%)	154 (8.6%)	
Smoking						
no	6120 (67.2%)	651 (77.2%)	<0.001	10,667 (96.0)	1735 (97.0%)	0.035
yes	2988 (32.8%)	192 (22.8%)		444 (4.0%)	53 (3.0%)	
Alcohol drinking						
low	7330 (80.5%)	777 (92.2%)	<0.001	10,671 (96.0%)	1766 (98.8%)	<0.001
high	1778 (19.5%)	66 (7.8%)		440 (4.0%)	22 (1.2%)	
Physical activity						
<150 min/week	4930 (54.1%)	620 (73.5%)	<0.001	6547 (58.9%)	1361 (76.1%)	<0.001
≥150 min/week	4178 (45.9%)	223 (26.5%)		4564 (41.1%)	427 (23.9%)	
Resistance exercise						
<2/week	6493 (71.3%)	732 (86.8%)	<0.001	9491 (85.4%)	1680 (94.0%)	<0.001
≥2/week	2615 (28.7%)	111 (13.2%)		1620 (14.6%)	108 (6.0%)	
Hypertension						
no	6050 (66.4%)	439 (52.1%)	<0.001	7982 (71.8%)	891 (49.8%)	<0.001
yes	3058 (33.6%)	404 (47.9%)		3129 (28.2%)	897 (50.2%)	
Diabetes						
no	7836 (86.0%)	651 (77.2%)	<0.001	10,072 (90.6%)	1407 (78.7%)	<0.001
yes	1272 (14.0%)	192 (22.8%)		1039 (9.4%)	381 (21.3%)	
Dyslipidemia						
no	7281 (79.9%)	689 (81.7%)	0.213	8320 (74.9%)	1196 (66.95)	<0.001
yes	1827 (20.1%)	154 (18.3%)		2791 (25.1%)	592 (33.1%)	
Ischemic heart disease						
no	8668 (95.2%)	752 (89.2%)	<0.001	10,846 (97.6%)	1688 (94.4%)	<0.001
yes	440 (4.8%)	91 (17.1%)		265 (2.4%)	100 (5.6%)	
Stroke						
no	8820 (96.8%)	747 (88.6%)	<0.001	10,887 (98.0%)	1669 (93.3%)	<0.001
yes	288 (3.2%)	96 (11.4%)		224 (2.0%)	119 (6.7%)	
Cancer						
no	8568 (94.1%)	760 (90.2%)	<0.001	10,286 (92.6%)	1652 (92.4%)	0.787
yes	540 (5.9%)	83 (9.8%)		825 (7.4%)	136 (7.6%)	
Arthritis						
no	8469 (93.0%)	711 (84.3%)	<0.001	8643 (77.8%)	1080 (60.4%)	<0.001
yes	639 (7.0%)	132 (15.7%)		2468 (22.2%)	708 (39.6%)	

**Table 2:** Sex-specific logistic regression for dynapenia: odds ratios (ORs) with 95% confidence intervals (CIs) from univariable and multivariable models.

	Men			Women		
	Univariable OR (95%CI)	Model 1 OR (95%CI)	Model 2 OR (95%CI)	Univariable OR (95%CI)	Model 1 OR (95%CI)	Model 2 OR (95%CI)
Age						
≥75	33.23 *** (26.11–42.29)	-	16.90 *** (12.80–22.30)	16.06 *** (13.92–18.52)	-	8.88 *** (7.41–10.63)
60–74	5.02 *** (3.93–6.41)	-	3.51 *** (2.69–4.58)	3.09 *** (2.70–3.54)	-	2.15 *** (1.84–2.52)
40–59	1.0 (reference)	1.0 (reference)	1.0 (reference)	1.0 (reference)	1.0 (reference)	1.0 (reference)
Household income						
high	0.08 *** (0.06–0.11)	0.27 *** (0.20–0.35)	0.35 *** (0.26–0.47)	0.18 *** (0.15–0.21)	0.51 *** (0.43–0.61)	0.59 *** (0.49–0.70)
middle-high	0.16 *** (0.13–0.20)	0.41 *** (0.32–0.52)	0.49 *** (0.38–0.62)	0.24 *** (0.21–0.28)	0.57 *** (0.48–0.67)	0.61 *** (0.52–0.73)
middle-low	0.32 *** (0.27–0.39)	0.57 *** (0.47–0.69)	0.62 *** (0.51–0.76)	0.38 *** (0.33–0.43)	0.70 *** (0.60–0.80)	0.72 *** (0.63–0.84)
low	1.0 (reference)	1.0 (reference)	1.0 (reference)	1.0 (reference)	1.0 (reference)	1.0 (reference)
Education						
≥college	0.25 *** (0.20–0.31)	0.46 *** (0.37–0.58)	0.69** (0.54–0.88)	0.29 *** (0.24–0.34)	0.66 *** (0.55–0.79)	0.78 * (0.64–0.95)
<college	1.0 (reference)	1.0 (reference)	1.0 (reference)	1.0 (reference)	1.0 (reference)	1.0 (reference)
Smoking						
yes	0.60 *** (0.51–0.71)	1.13 (0.94–1.36)	0.98 (0.81–1.19)	0.73 * (0.55–0.98)	1.05 (0.77–1.43)	0.96 (0.69–1.32)
no	1.0 (reference)	1.0 (reference)	1.0 (reference)	1.0 (reference)	1.0 (reference)	1.0 (reference)
Alcohol drinking						
high	0.35 *** (0.27–0.45)	0.69 ** (0.52–0.91)	0.69 ** (0.52–0.91)	0.30 *** (0.20–0.47)	0.56 * (0.36–0.88)	0.53** (0.34–0.84)
low	1.0 (reference)	1.0 (reference)	1.0 (reference)	1.0 (reference)	1.0 (reference)	1.0 (reference)
Physical activity						
≥150 min/week	0.42 *** (0.36–0.50)	0.53 *** (0.45–0.63)	0.63 *** (0.53–0.75)	0.45 *** (0.40–0.51)	0.63 *** (0.56–0.72)	0.68 *** (0.60–0.78)
<150 min/week	1.0 (reference)	1.0 (reference)	1.0 (reference)	1.0 (reference)	1.0 (reference)	1.0 (reference)
Resistance exercise						
≥2/week	0.38 *** (0.31–0.46)	0.39 *** (0.31–0.49)	0.47 *** (0.38–0.59)	0.38 *** (0.31–0.46)	0.51 *** (0.41–0.62)	0.58 *** (0.47–0.71)
<2/week	1.0 (reference)	1.0 (reference)	1.0 (reference)	1.0 (reference)	1.0 (reference)	1.0 (reference)
Hypertension						
yes	1.82 *** (1.58–2.10)	0.92 (0.78–1.07)	0.83 * (0.70–0.98)	2.57 *** (2.32–2.84)	1.08 (0.96–1.21)	0.92 (0.81–1.04)
no	1.0 (reference)	1.0 (reference)	1.0 (reference)	1.0 (reference)	1.0 (reference)	1.0 (reference)
Diabetes						
yes	1.82 *** (1.53–2.16)	1.27 * (1.05–1.53)	1.23 * (1.00–1.50)	2.63 *** (2.31–3.00)	1.49 *** (1.29–1.72)	1.39 *** (1.20–1.62)
no	1.0 (reference)	1.0 (reference)	1.0 (reference)	1.0 (reference)	1.0 (reference)	1.0 (reference)
Dyslipidemia						
yes	0.89 (0.74–1.07)	0.85 (0.70–1.03)	0.85 (0.69–1.05)	1.48 *** (1.33–1.64)	1.05 (0.94–1.19)	0.98 (0.87–1.11)
no	1.0 (reference)	1.0 (reference)	1.0 (reference)	1.0 (reference)	1.0 (reference)	1.0 (reference)
Ischemic heart disease						
yes	2.38 *** (1.88–3.02)	1.30 (1.00–1.68)	1.24 (0.95–1.62)	2.43 *** (1.92–3.07)	1.30 * (1.01–1.70)	1.23 (0.95–1.59)
no	1.0 (reference)	1.0 (reference)	1.0 (reference)	1.0 (reference)	1.0 (reference)	1.0 (reference)
Stroke						
yes	3.94 *** (3.09–5.02)	2.32 *** (1.76–3.04)	2.06 *** (1.55–2.73)	3.47 *** (2.76–4.35)	1.77 *** (1.37–2.28)	1.58 *** (1.23–2.05)
no	1.0 (reference)	1.0 (reference)	1.0 (reference)	1.0 (reference)	1.0 (reference)	1.0 (reference)
Cancer						
yes	1.73 *** (1.36–2.21)	0.92 (0.71–1.20)	0.90 (0.69–1.18)	1.03 (0.85–1.24)	0.96 (0.78–1.18)	0.98 (0.79–1.20)
no	1.0 (reference)	1.0 (reference)	1.0 (reference)	1.0 (reference)	1.0 (reference)	1.0 (reference)
Arthritis						
yes	2.46 *** (2.01–3.01)	1.35** (1.08–1.69)	1.21 (0.96–1.53)	2.30 *** (2.07–2.55)	1.24 *** (1.10–1.39)	1.16 * (1.03–1.31)
no	1.0 (reference)	1.0 (reference)	1.0 (reference)	1.0 (reference)	1.0 (reference)	1.0 (reference)

Abbreviations: OR, odds ratio; CI, confidence interval. Model 1: adjusted for age. Model 2: adjusted for age, household income, education, smoking, alcohol drinking, physical activity, resistance exercise, hypertension, diabetes, dyslipidemia, ischemic heart disease, stroke, cancer, and arthritis. * *p* < 0.05, ** *p* < 0.01, *** *p* < 0.001.

**Table 3:** Top five association rules for dynapenia by sex, identified through Association Rule Mining.

Rules	Support	Confidence	Coverage	Lift	Count
Association rules for men					
{physical activity < 150 min/week, age ≥ 75 year, low-risk drinking, alcohol, diabetes, education ≤ high school, normotension, resistance exercise < 2/week} **→** {with dynapenia}	0.0025	0.5435	0.00462	6.4154	25
{physical activity < 150 min/week, age ≥ 75 year, diabetes, education ≤ high school, normotension, low household income, resistance exercise < 2/week} **→** {with dynapenia}	0.0017	0.5152	0.00332	6.0810	17
{age ≥ 75 year, low-risk drinking, diabetes, education ≤ high school, normotension, low household income, resistance exercise < 2/week} **→** {with dynapenia}	0.0017	0.5152	0.00332	6.0810	17
{physical activity < 150 min/week, age ≥ 75 year, low-risk drinking, diabetes, normotension, low household income, resistance exercise < 2/week} **→** {with dynapenia}	0.0017	0.5000	0.00342	5.9021	17
{physical activity < 150 min/week, age ≥ 75 year, low-risk drinking, education ≤ high school, normotension, low household income, resistance exercise < 2/week} **→** {with dynapenia}	0.0121	0.4959	0.02432	5.8534	120
Association rules for women					
{physical activity < 150 min/week, age ≥ 75 year, low-risk drinking, diabetes, normotension, low household income, resistance exercise < 2/week} **→** {with dynapenia}	0.0023	0.5882	0.0040	4.2437	30
{physical activity < 150 min/week, age ≥ 75 year, low-risk drinking, diabetes, education ≤ high school, normotension, low household income} **→** {with dynapenia}	0.0024	0.5849	0.0041	4.2196	31
{physical activity < 150 min/week, age ≥ 75 year, diabetes, education ≤ high school, normotension, low household income, resistance exercise < 2/week} **→** {with dynapenia}	0.0022	0.5800	0.0039	4.1842	29
{age ≥ 75 year, low-risk drinking, diabetes, education ≤ high school, normotension, low household income, resistance exercise < 2/week} **→** {with dynapenia}	0.0022	0.5800	0.0039	4.1842	29
{physical activity < 150 min/week, age ≥ 75 year, low-risk drinking, absence of diabetes, normotension, low-middle household income, resistance exercise < 2/week} **→** {with dynapenia }	0.0015	0.5429	0.0027	3.9163	19

## Data Availability

The data presented in this study are openly available in the KNHANES database and can be downloaded after obtaining institutional approval from the KNHANES database administrators.

## Supplementary Materials

The following supporting information can be downloaded at: https://www.mdpi.com/article/10.3390/jpm15110507/s1, Table S1: Summary statistics before and after listwise deletion; Table S2: Sex-specific descriptive characteristics of participants according to dynapenia status with standardized differences; Table S3: Sex-specific complex-sample logistic regression analysis for dynapenia; Table S4: Dichotomization codebook; Table S5: Algorithm parameters for ARM; Table S6: Sex-specific multivariable logistic regression for dynapenia defined by the dominant hand; Table S7: Example of Top Rules Output. ARM Association Rule Mining AWGS Asian Working Group for Sarcopenia KNHANES  Korea National Health and Nutrition Examination Survey WHO World Health Organization.

## Abbreviations

The following abbreviations are used in this manuscript:

ARM	Association Rule Mining
AWGS	Asian Working Group for Sarcopenia
KNHANES	Korea National Health and Nutrition Examination Survey
WHO	World Health Organization
