# Supplementary material for: Sex-Specific Risk Factors for Dynapenia in Korean Middle-Aged and Older Adults: A Cross-Sectional Study Based on the Korea National Health and Nutrition Examination Survey 2014–2019
Source: J Pers Med. 2025 Oct 25;15(11):507. doi: 10.3390/jpm15110507 (PMC12653596; doi:10.3390/jpm15110507)
Supplement: Supplementary file 1 [file jpm-15-00507-s001.zip › jpm-3904818-supplementary.pdf]

Supplemental Materials

Supplementary Table S1. Summary statistics before and after listwise deletion

Supplementary Table S2. Sex-specific descriptive characteristics of participants according to dynapenia status with standardized differences.

Supplementary Table S3. Sex-specific complex-sample logistic regression analysis for dynapenia

Supplementary Table S4. Dichotomization codebook

Supplementary Table S5. Algorithm parameters for ARM

Supplementary Table S6. Sex-specific multivariable logistic regression for dynapenia defined by the dominant hand

Supplementary Table S7. Example of Top Rules Output

Supplementary Table S1. Summary statistics before and after listwise deletion

	Raw data		Analysis dataset		P-value
Sex					
men	10,863	(43.9%)	9,951	(43.5%)	0.396
women	13,862	(56.1%)	12,899	(56.5%)	
Age					
40-59	12,787	(51.7%)	11,963	(52.4%)	0.011
60-74	8,445	(34.2%)	7,876	(34.5%)	
≥ 75	3,493	(14.1%)	3,011	(13.2%)	
Household income					
low	5,796	(23.6%)	5,195	(22.7%)	0.170
middle-low	6,147	(25.0%)	5,713	(25.0%)	
middle-high	6014	(24.4%)	5,651	(24.7%)	
high	6,646	(27.0%)	6,291	(27.5%)	
Education					
< college	16,756	(72.5%)	16,532	(72.4%)	0.671
≥ college	6,347	(27.5%)	6,318	(27.6%)	
Smoking					
no	20,133	(83.7%)	19,173	(83.9%)	0.480
yes	3,930	(16.3%)	3,677	(16.1%)	
Alcohol drinking					
low	21,650	(89.8%)	20,544	(89.9%)	0.790
high	2,450	(10.2%)	2,306	(10.1%)	
Physical activity					
<150min	13,691	(59.1%)	13,458	(58.9%)	0.700
≥150min	9,485	(40.9%)	9,392	(41.1%)	
Resistance exercise					
< 2/week	18,646	(80.6%)	18,396	(80.5%)	0.824
≥ 2/week	4,491	(19.4%)	4,454	(19.5%)	
Hypertension					
no	16,142	(67.0%)	15,362	(67.2%)	0.653
yes	7,938	(33.0%)	7,488	(32.8%)	
Diabetes					
no	21,007	(87.3%)	19,966	(87.4%)	0.726
yes	3,064	(12.7%)	2,884	(12.6%)	
Dyslipidemia					
no	18,461	(76.7%)	17,486	(76.5%)	0.714
yes	5,618	(23.3%)	5,364	(23.5%)	
Ischemic heart disease					
no	22,342	(96.0%)	21,954	(96.1%)	0.817
yes	922	(4.0%)	896	(3.9%)	
Stroke					
no	22,528	(96.8%)	22,123	(96.8%)	0.825
yes	749	(3.2%)	727	(3.2%)	
Cancer					
no	21,646	(93.1%)	21,266	(93.1%)	0.969
yes	1,610	(6.9%)	1,584	(6.9%)	
Arthritis					
no	19,221	(82.6%)	18,903	(82.7%)	0.773
yes	4,042	(17.4%)	3,947	(17.3%)	

Supplementary Table S2. Sex-specific descriptive characteristics of participants according to dynapenia status with standardized differences.

	**Men** (*N*=9951)				**Women** (*N*=12,899)			
	**Without dynapenia** (*n*=9108)	**Dynapenia** (*n*=843)	**P value**	**SD**	**Without dynapenia** (*n*=11,111)	**Dynapenia** (*n*=1788)	**P value**	**SD**
**Age**								
40-59	5005(55.0%)	86(10.2%)	<0.001	1.39	6513(58.6%)	359(20.1%)	<0.001	1.07
60-74	3271(35.9%)	282(33.5%)			3694(33.2%)	629(35.2%)		
≥ 75	832(9.1%)	475(56.3%)			904(8.1%)	800(44.7%)		
**Household income**								
low	1551(17.0%)	448(53.1%)	<0.001	0.95	2302(20.7%)	894(28.0%)	<0.001	0.69
middle-low	2301(25.3%)	215(25.5%)			2790(25.1%)	407(22.8%)		
middle-high	2438(26.8%)	113(13.4%)			2835(25.5%)	265(14.8%)		
high	2818(30.9%)	67(7.9%)			3184(28.7%)	222(12.4%)		
**Education**								
< college	5807(63.8%)	738(87.5%)	<0.001	0.58	8353(75.2%)	1634(91.4%)	<0.001	0.45
≥ college	3301(36.2%)	105(12.5%)			2758(24.8%)	154(8.6%)		
**Smoking**								
no	6120(67.2%)	651(77.2%)	<0.001	0.23	10667(96.0)	1735(97.0%)	0.035	0.06
yes	2988(32.8%)	192(22.8%)			444(4.0%)	53(3.0%)		
**Alcohol drinking**								
low	7330(80.5%)	777(92.2%)	<0.001	0.35	10671(96.0%)	1766(98.8%)	<0.001	0.17
high	1778(19.5%)	66(7.8%)			440(4.0%)	22(1.2%)		
**Physical activity**								
<150min/week	4930(54.1%)	620(73.5%)	<0.001	0.41	6547(58.9%)	1361(76.1%)	<0.001	0.37
≥150min/week	4178(45.9%)	223(26.5%)			4564(41.1%)	427(23.9%)		
**Resistance exercise**								
< 2/week	6493(71.3%)	732(86.8%)	<0.001	0.39	9491(85.4%)	1680(94.0%)	<0.001	0.28
≥ 2/week	2615(28.7%)	111(13.2%)			1620(14.6%)	108(6.0%)		
**Hypertension**								
no	6050(66.4%)	439(52.1%)	<0.001	0.30	7982(71.8%)	891(49.8%)	<0.001	0.46

yes	3058(33.6%)	404(47.9%)			3129(28.2%)	897(50.2%)		
**Diabetes**								
no	7836(86.0%)	651(77.2%)	<0.001	0.23	10072(90.6%)	1407(78.7%)	<0.001	0.34
yes	1272(14.0%)	192(22.8%)			1039(9.4%)	381(21.3%)		
**Dyslipidemia**								
no	7281(79.9%)	689(81.7%)	0.213	0.05	8320(74.9%)	1196(66.95)	<0.001	0.18
yes	1827(20.1%)	154(18.3%)			2791(25.1%)	592(33.1%)		
**Ischemic heart disease**								
no	8668(95.2%)	752(89.2%)	<0.001	0.22	10846(97.6%)	1688(94.4%)	<0.001	0.16
yes	440(4.8%)	91(17.1%)			265(2.4%)	100(5.6%)		
**Stroke**								
no	8820(96.8%)	747(88.6%)	<0.001	0.32	10887(98.0%)	1669(93.3%)	<0.001	0.23
yes	288(3.2%)	96(11.4%)			224(2.0%)	119(6.7%)		
**Cancer**								
no	8568(94.1%)	760(90.2%)	<0.001	0.15	10286(92.6%)	1652(92.4%)	0.787	0.01
yes	540(5.9%)	83(9.8%)			825(7.4%)	136(7.6%)		
**Arthritis**								
no	8469(93.0%)	711(84.3%)	<0.001	0.28	8643(77.8%)	1080(60.4%)	<0.001	0.38
yes	639(7.0%)	132(15.7%)			2468(22.2%)	708(39.6%)		

Data are presented as n (%). P-values were calculated using chi-squared tests. SD, standardized difference; SD values >0.1 indicate meaningful imbalance between groups.

Supplementary Table S3. Sex-specific complex-sample logistic regression analysis for dynapenia

	Men			Women		
	**Univariable OR (95%CI)**	**Model 1 OR (95%CI)**	**Model 2 OR (95%CI)**	**Univariable OR (95%CI)**	**Model 1 OR (95%CI)**	**Model 2 OR (95%CI)**
**Age**						
≥ 75	38.52***(29.69-49.99)	-	17.16***(12.21-24.14)	18.19***(15.38-21.50)	-	8.60***(6.91-10.70)
60-74	7.44***(6.02-8.93)	-	4.87***(3.92-6.03)	6.16***(5.39-7.04)	-	4.44***(3.80-5.17)
40-59	1.0(reference)	1.0(reference)	1.0(reference)	1.0(reference)	1.0(reference)	1.0(reference)
**Household income**						
high	0.08***(0.06-0.10)	0.27***(0.20-0.35)	0.26***(0.18-0.36)	0.16***(0.13-0.19)	0.49***(0.40-0.60)	0.61***(0.49-0.75)
middle-high	0.27***(0.19-0.37)	0.41***(0.32-0.52)	0.47***(0.33-0.67)	0.45***(0.37-0.55)	0.69***(0.58-0.85)	0.82***(0.67-1.02)
middle-low	0.59**(0.42-0.83)	0.70*(0.50-0.69)	0.78*(0.54-1.00)	0.75**(0.61-0.93)	0.91(0.74-1.13)	1.01(0.81-1.26)
low	1.0(reference)	1.0(reference)	1.0(reference)	1.0(reference)	1.0(reference)	1.0(reference)
**Education**						
≥ college	0.28***(0.22-0.35)	0.52***(0.40-0.68)	0.80(0.60-1.07)	0.28***(0.23-0.35)	0.63***(0.50-0.79)	0.72**(0.57-0.92)
< college	1.0(reference)	1.0(reference)	1.0(reference)	1.0(reference)	1.0(reference)	1.0(reference)
**Smoking**						
yes	0.59***(0.49-0.70)	1.08(0.88-1.32)	0.99(0.79-1.24)	0.64**(0.46-0.89)	0.86(0.61-1.22)	0.88(0.62-1.26)
no	1.0(reference)	1.0(reference)	1.0(reference)	1.0(reference)	1.0(reference)	1.0(reference)
**Alcohol drinking**						
high	0.34***(0.29-0.40)	0.50***(0.42-0.60)	0.56***(0.45-0.68)	0.40***(0.35-0.46)	0.70***(0.60-0.80)	0.71***(0.61-0.84)
low	1.0(reference)	1.0(reference)	1.0(reference)	1.0(reference)	1.0(reference)	1.0(reference)
**Physical activity**						
≥150min/week	0.41***(0.34-0.49)	0.52***(0.42-0.63)	0.63***(0.51-0.78)	0.44***(0.38-0.50)	0.64***(0.56-0.74)	0.69***(0.60-0.80)
<150min/week	1.0(reference)	1.0(reference)	1.0(reference)	1.0(reference)	1.0(reference)	1.0(reference)
**Resistance exercise**						
≥ 2/week	0.38***(0.30-0.48)	0.39***(0.31-0.50)	0.49***(0.38-0.63)	0.41***(0.32-0.52)	0.55***(0.42-0.71)	0.63***(0.49-0.81)
< 2/week	1.0(reference)	1.0(reference)	1.0(reference)	1.0(reference)	1.0(reference)	1.0(reference)
**Hypertension**						
yes	2.04***(1.74-2.40)	0.93(0.78-1.12)	0.83*(0.70-0.98)	2.91***(2.60-3.27)	1.13(0.99-1.30)	1.01(0.87-1.17)
no	1.0(reference)	1.0(reference)	1.0(reference)	1.0(reference)	1.0(reference)	1.0(reference)
**Diabetes**						
yes	2.11***(1.75-2.55)	1.32*(1.07-1.64)	1.23*(1.00-1.50)	2.93***(2.55-3.38)	1.54***(1.30-1.83)	1.38***(1.15-1.66)
no	1.0(reference)	1.0(reference)	1.0(reference)	1.0(reference)	1.0(reference)	1.0(reference)
**Dyslipidemia**						
yes	0.95(0.78-1.17)	0.85(0.68-1.06)	0.85(0.69-1.05)	1.48***(1.31-1.66)	1.01(0.88-1.17)	0.97(0.82-1.13)
no	1.0(reference)	1.0(reference)	1.0(reference)	1.0(reference)	1.0(reference)	1.0(reference)
**Ischemic heart disease**						
yes	2.86***(2.17-3.78)	1.38(0.99-1.91)	1.24(0.95-1.62)	2.32***(1.79-3.02)	1.09(0.81-1.47)	1.05(0.78-1.43)
no	1.0(reference)	1.0(reference)	1.0(reference)	1.0(reference)	1.0(reference)	1.0(reference)
**Stroke**						
yes	5.32***(4.01-7.06)	2.75***(2.00-3.77)	2.06***(1.55-2.73)	3.41***(2.61-4.44)	1.56*(1.11-2.19)	1.36(0.97-1.93)

no	1.0(reference)	1.0(reference)	1.0(reference)	1.0(reference)	1.0(reference)	1.0(reference)
**Cancer**						
yes	1.91***(1.45-2.53)	0.85(0.63-1.15)	0.90(0.69-1.18)	1.07(0.86-1.33)	0.98(0.77-1.25)	0.95(0.74-1.21)
no	1.0(reference)	1.0(reference)	1.0(reference)	1.0(reference)	1.0(reference)	1.0(reference)
**Arthritis**						
yes	2.86***(2.29-3.58)	1.43*(1.09-1.88)	1.21(0.96-1.53)	2.47***(2.20-2.78)	1.20**(1.05-1.37)	1.12(0.97-1.29)
no	1.0(reference)	1.0(reference)	1.0(reference)	1.0(reference)	1.0(reference)	1.0(reference)

Abbreviations: OR, odds ration; CI, confidence interval

Model 1: adjusted for age, Model 2: adjusted for age, household income, education, smoking, alcohol drinking, physical activity, resistance exercise, hypertension, diabetes, dyslipidemia, ischemic heart disease, stroke, cancer, and arthritis.

*P < 0.05, **P < 0.01, ***P < 0.001

Supplementary Table S4. Dichotomization codebook

Variable name	Dichotomization criteria	Coding used in ARM	Notes
Dynapenia	0 = without dynapenia<br>1 = with dynapenia	1 = with dynapenia	AWGS2019 criteria<br>Maximum grip strength of both hands
Age	1 = 40-59<br>2 = 60-74<br>3 = ≥75	1 = age 40-59year<br>2 = age 60-74year<br>3 = age≥75year	
Household income	1 = low<br>2 = mid-low<br>3 = mid-high<br>4 = high	1 = low household income<br>2 = low-middle household income<br>3 = middle-high household income<br>4 = high household income	Household income quartile
Education	1 ≤ high school<br>2 ≥ college	1 ≤ high school<br>2 ≥ college	
Smoking	1 = no<br>2 = yes	1 = non-smoker<br>2 = current smoker	
Alcohol drinking	1 = low<br>2 = high	1 = low-risk drinking<br>2 = high-risk drinking	
Physical activity	1 < 150min/week<br>2 ≥150min/week	1 physical activity<150min/week<br>2 physical activity≥150min/week	WHO recommendation<br>Based on IPAQ
Resistance exercise	1 <2/week<br>2 ≥2/week	1 resistance exercise<2/week<br>2 resistance exercise≥2/week	WHO recommendation
Hypertension	0 = No<br>1 = Yes	0 = normotension<br>1 = hypertension	Physician diagnosis
Diabetes	0 = No<br>1 = Yes	0 = absence of diabetes<br>1 = diabetes	Physician diagnosis
Dyslipidemia	0 = No<br>1 = Yes	0 = no dyslipidemia<br>1 = dyslipidemia	Physician diagnosis
Ischemic heart disease	0 = No<br>1 = Yes	0 = no ischemic heart disease<br>1 = ischemic heart disease	Physician diagnosis
Stroke	0 = No<br>1 = Yes	0 = no stroke<br>1 = stroke	Physician diagnosis
Cancer	0 = No<br>1 = Yes	0 = no cancer<br>1 = cancer	Physician diagnosis
Arthritis	0 = No<br>1 = Yes	0 = no arthritis<br>1 = arthritis	Physician diagnosis

Supplementary Table S5. Algorithm parameters for ARM

Parameter	Value used in this study	Description
Minimum support	0.001	Minimum proportion of records containing an itemset. A low threshold was applied to capture rare but potentially meaningful associations, given the large sample size.
Minimum confidence	0.1	Minimum conditional probability that the consequent occurs when the antecedent is present. This liberal cutoff allowed exploratory pattern discovery.
Minimum rule length (minlen)	8	Minimum number of items (antecedent + consequent) per rule. Fixed at 8 to focus on complex interaction structures.
Maximum rule length (maxlen)	8	Maximum number of item allowed per rule. Set equal to minlen (8) to restrict rules to exactly 8 items for interpretability and computational efficiency.
Evaluation metrics	Support, Confidence, Lift, Coverage	Rules were primarily ranked by descending lift values, with lift > 1 considered meaningful

Supplementary Table S6. Sex-specific multivariable logistic regression for dynapenia defined by the dominant hand

	Men	Women
	**OR (95%CI)**	**OR (95%CI)**
**Age**		
≥ 75	13.52***(10.60-17.24)	8.19***(6.90-9.72)
60-74	3.31***(2.64-4.15)	2.15***(1.86-2.49)
40-59	1.0(reference)	1.0(reference)
**Household income**		
high	0.38***(0.29-0.49)	0.63***(0.54-0.75)
middle-high	0.50***(0.40-0.62)	0.64***(0.55-0.75)
middle-low	0.60***(0.50-0.72)	0.71***(0.62-0.81)
low	1.0(reference)	1.0(reference)
**Education**		
≥ college	070***(0.60-1.07)	0.91(0.77-1.08)
< college	1.0(reference)	1.0(reference)
**Smoking**		
yes	1.03(0.87-1.22)	0.96(0.71-1.23)
no	1.0(reference)	1.0(reference)
**Alcohol drinking**		
high	0.63***(0.49-0.81)	0.72(0.49-1.04)
low	1.0(reference)	1.0(reference)
**Physical activity**		
≥150min/week	0.65***(0.55-0.76)	0.67***(0.60-0.76)
<150min/week	1.0(reference)	1.0(reference)
**Resistance exercise**		
≥ 2/week	0.50***(0.41-0.61)	0.59***(0.49-0.72)
< 2/week	1.0(reference)	1.0(reference)
**Hypertension**		
yes	0.93(0.79-1.08)	0.94(0.83-1.06)
no	1.0(reference)	1.0(reference)
**Diabetes**		
yes	1.23*(1.01-1.45)	1.38***(1.19-1.59)
no	1.0(reference)	1.0(reference)
**Dyslipidemia**		
yes	0.86(0.71-1.04)	0.95(0.84-1.08)
no	1.0(reference)	1.0(reference)
**Ischemic heart disease**		
yes	1.04(0.80-1.35)	1.16(0.90-1.50)
no	1.0(reference)	1.0(reference)
**Stroke**		
yes	1.77***(1.34-2.32)	1.58***(1.23-2.04)
no	1.0(reference)	1.0(reference)
**Cancer**		
yes	0.91(0.70-1.17)	1.01(0.83-1.23)
no	1.0(reference)	1.0(reference)
**Arthritis**		
yes	1.39**(1.12-1.72)	1.21**(1.08-1.36)
no	1.0(reference)	1.0(reference)

Abbreviations: OR, odds ration; CI, confidence interval

Adjusted for age, household income, education, smoking, alcohol drinking, physical activity, resistance exercise, hypertension, diabetes, dyslipidemia, ischemic heart disease, stroke, cancer, and arthritis.

$^{*}P < 0.05$, $^{**}P < 0.01$, $^{***}P < 0.001$

Supplementary Table S7. Example of Top Rules Output

Rules	Support	Confidence	Coverage	Lift
**Association rules for men**				
{physical activity<150min/week, age≥75year, low-risk drinking, diabetes, education≤high school, normotension, resistance exercise<2/week} → {with dynapenia}	0.0025	0.5435	0.0046	6.4154
{physical activity<150min/week, age≥75year, diabetes, education≤high school, normotension, low household income, resistance exercise<2/week} → {with dynapenia}	0.0017	0.5152	0.0033	6.0810
{age≥75year, low-risk drinking, diabetes, education≤high school, normotension, low household income, resistance exercise<2/week} → {with dynapenia}	0.0017	0.5152	0.0033	6.0810
{physical activity<150min/week, age≥75year, low-risk drinking, diabetes, normotension, low household income, resistance exercise<2/week} → {with dynapenia}	0.0017	0.5000	0.0034	5.9021
{physical activity<150min/week, age≥75year, low-risk drinking, education≤high school, normotension, low household income, resistance exercise<2/week} → {with dynapenia}	0.0121	0.4959	0.0243	5.8534
{physical activity<150min/week, age≥75year, absence of diabetes, education≤high school, normotension, low household income, resistance exercise<2/week} → {with dynapenia}	0.0104	0.4928	0.0210	5.8174
{age≥75year, low-risk drinking, absence of diabetes, education≤high school, normotension, low household income, resistance exercise<2/week} → {with dynapenia}	0.0104	0.4928	0.0210	5.8174
{physical activity<150min/week, age≥75year, low-risk drinking, absence of diabetes, normotension, low household income, resistance exercise<2/week} → {with dynapenia}	0.0105	0.4749	0.0220	5.6057
{physical activity<150min/week, age≥75year, low-risk drinking, absence of diabetes, education≤high school, normotension, low household income} → {with dynapenia}	0.0115	0.4691	0.0244	5.5378
{physical activity<150min/week, age≥75year, low-risk drinking, absence of diabetes, education≤high school, normotension, resistance exercise<2/week} → {with dynapenia}	0.0153	0.4677	0.0327	5.5208
{physical activity<150min/week, age≥75year, low-risk drinking, diabetes, education≤high school, low household income, resistance exercise<2/week} → {with dynapenia}	0.0049	0.4667	0.0106	5.5087

{physical activity<150min/week, age≥75year, low-risk drinking, education≤high school, normotension, low-middle household income, resistance exercise<2/week} → {with dynapenia}	0.0039	0.4588	0.0085	5.4161
{physical activity<150min/week, age≥75year, low-risk drinking, absence of diabetes, education≤high school, low household income, resistance exercise<2/week} → {with dynapenia}	0.0196	0.4556	0.0430	5.3781
{physical activity<150min/week, age≥75year, low-risk drinking, diabetes, education≤high school, normotension, low household income} → {with dynapenia}	0.0018	0.4500	0.0040	5.3119
{physical activity<150min/week, age≥75year, diabetes, education≤high school, hypertension, low household income, resistance exercise<2/week} → {with dynapenia}	0.0032	0.4444	0.0072	5.2463
{age≥75year, low-risk drinking, diabetes, education≤high school, hypertension, low household income, resistance exercise<2/week} → {with dynapenia}	0.0032	0.4444	0.0072	5.2463
{physical activity<150min/week, age≥75year, absence of diabetes, education≤high school, normotension, low-middle household income, resistance exercise<2/week} → {with dynapenia}	0.0033	0.4400	0.0075	5.1939
{age≥75year, low-risk drinking, absence of diabetes, education≤high school, normotension, low-middle household income, resistance exercise<2/week} → {with dynapenia}	0.0033	0.4400	0.0075	5.1939
{physical activity<150min/week, age≥75year, absence of diabetes, education≤high school, normotension, low-middle household income, resistance exercise<2/week} → {with dynapenia},	0.0019	0.4318	0.0044	5.0973
{physical activity<150min/week, age≥75year, low-risk drinking, education≤high school, hypertension, low household income, resistance exercise<2/week} → {with dynapenia}	0.0125	0.4261	0.0292	5.0300
**Association rules for women**				
{physical activity<150min/week, age≥75year, low-risk drinking, diabetes, normotension, low household income, resistance exercise<2/week} → {with dynapenia}	0.0023	0.5882	0.0040	4.2437
{physical activity<150min/week, age≥75year, low-risk drinking, diabetes, education≤high school, normotension, low household income} → {with dynapenia}	0.0024	0.5849	0.0041	4.2196
{physical activity<150min/week, age≥75year, diabetes, education≤high school, normotension, low household income, resistance exercise<2/week} → {with dynapenia}	0.0022	0.5800	0.0039	4.1842

{age≥75year, low-risk drinking, diabetes, education≤high school, normotension, low household income, resistance exercise<2/week} → {with dynapenia}	0.0022	0.5800	0.0039	4.1842
{physical activity<150min/week, age≥75year, low-risk drinking, absence of diabetes, normotension, middle-high household income, resistance exercise<2/week} → {with dynapenia}	0.0015	0.5429	0.0027	3.9163
{physical activity<150min/week, age≥75year, low-risk drinking, diabetes, education≤high school, normotension, resistance exercise<2/week} → {with dynapenia}	0.0030	0.5417	0.0056	3.9077
{physical activity<150min/week, age≥75year, absence of diabetes, education≤high school, normotension, middle-high household income, resistance exercise<2/week} → {with dynapenia}	0.0014	0.5294	0.0026	3.8193
{age≥75year, low-risk drinking, absence of diabetes, education≤high school, normotension, middle-high household income, resistance exercise<2/week} → {with dynapenia}	0.0014	0.5294	0.0026	3.8193
{physical activity<150min/week, age≥75year, low-risk drinking, absence of diabetes, education≤high school, normotension, middle-high household income} → {with dynapenia}	0.0015	0.5278	0.0028	3.8075
{physical activity<150min/week, age≥75year, low-risk drinking, education≤high school, normotension, middle-high household income, resistance exercise<2/week} → {with dynapenia}	0.0016	0.5250	0.0031	3.7875
{physical activity<150min/week, age≥75year, low-risk drinking, education≤high school, hypertension, high household income, resistance exercise<2/week} → {with dynapenia}	0.0026	0.5238	0.0049	3.7789
{physical activity<150min/week, age≥75year, low-risk drinking, diabetes, education≤high school, low household income, resistance exercise<2/week} → {with dynapenia}	0.0107	0.5227	0.0205	3.7711
{physical activity<150min/week, age≥75year, low-risk drinking, education≤high school, normotension, low household income, resistance exercise<2/week} → {with dynapenia}	0.0131	0.5200	0.0252	3.7514
{physical activity<150min/week, age≥75year, low-risk drinking, diabetes, hypertension, low household income, resistance exercise<2/week} → {with dynapenia}	0.0086	0.5115	0.0168	3.6902
{physical activity<150min/week, age≥75year, diabetes, education≤high school, hypertension, low household income, resistance exercise<2/week} → {with dynapenia}	0.0085	0.5093	0.0166	3.6745
{age≥75year, low-risk drinking, diabetes, education≤high school, hypertension, low household income, resistance exercise<2/week} → {with dynapenia}	0.0085	0.5093	0.0166	3.6745

{physical activity<150min/week, age≥75year, absence of diabetes, education≤high school, normotension, low household income, resistance exercise<2/week} → {with dynapenia}	0.0109	0.5091	0.0213	3.6727
{age≥75year, low-risk drinking, absence of diabetes, education≤high school, normotension, low household income, resistance exercise<2/week} → {with dynapenia}	0.0109	0.5091	0.0213	3.6727
{physical activity<150min/week, age≥75year, low-risk drinking, absence of diabetes, normotension, low household income, resistance exercise<2/week} → {with dynapenia}	0.0109	0.5054	0.0216	3.6459
{physical activity<150min/week, age≥75year, low-risk drinking, absence of diabetes, education≤high school, low household income, resistance exercise<2/week} → {with dynapenia}	0.0300	0.5019	0.0598	3.6211
